# Monitoring of T790M in plasma ctDNA of advanced EGFR-mutant NSCLC patients on first- or second-generation tyrosine kinase inhibitors

**DOI:** 10.1186/s12885-023-10698-5

**Published:** 2023-03-13

**Authors:** Chun-Ta Huang, Chih-An Lin, Te-Jen Su, Ching-Yao Yang, Tzu-Hsiu Tsai, Chia-Lin Hsu, Wei-Yu Liao, Kuan-Yu Chen, Chao-Chi Ho, Chong-Jen Yu

**Affiliations:** 1 Department of Internal Medicine, National Taiwan University Hospital, No. 7 Chung-Shan South Rd, Taipei 100, Taipei, Taiwan; 2 Graduate Institute of Clinical Medicine, National Taiwan University, Taipei, Taiwan; 3 Centers of Genomic and Precision Medicine, National Taiwan University, Taipei, Taiwan

**Keywords:** Droplet digital polymerase chain reaction, EGFR mutation, Lung cancer, Osimertinib, Tyrosine kinase inhibitor

## Abstract

**Background:**

The T790M mutation is the major resistance mechanism to first- and second-generation TKIs in EGFR-mutant NSCLC. This study aimed to investigate the utility of droplet digital PCR (ddPCR) for detection of T790M in plasma circulating tumor DNA (ctDNA), and explore its impact on prognosis.

**Methods:**

This prospective study enrolled 80 advanced lung adenocarcinoma patients treated with gefitinib, erlotinib, or afatinib for TKI-sensitizing mutations between 2015 and 2019. Plasma samples were collected before TKI therapy and at tri-monthly intervals thereafter. Genotyping of ctDNA for T790M was performed using a ddPCR EGFR Mutation Assay. Patients were followed up until the date of death or to the end of 2021.

**Results:**

Seventy-five of 80 patients experienced progressive disease. Fifty-three (71%) of 75 patients underwent rebiopsy, and T790M mutation was identified in 53% (28/53) of samples. Meanwhile, plasma ddPCR detected T790M mutation in 23 (43%) of 53 patients. The concordance rate of T790M between ddPCR and rebiopsy was 76%, and ddPCR identified 4 additional T790M-positive patients. Ten (45%) of 22 patients who did not receive rebiopsy tested positive for T790M by ddPCR. Serial ddPCR analysis showed the time interval from detection of plasma T790M to objective progression was 1.1 (0–4.1) months. Compared to 28 patients with rebiopsy showing T790M, the overall survival of 14 patients with T790M detected solely by ddPCR was shorter(41.3 [95% CI, 36.6–46.0] vs. 26.6 months [95% CI, 9.9–43.3], respectively).

**Conclusion:**

Plasma ddPCR-based genotyping is a useful technology for detection and monitoring of the key actionable genomic alteration, namely, T790M, in patients treated with gefitinib, erlotinib, or afatinib for activating mutations, to achieve better patient care and outcome.

## Introduction

Lung cancer is the most prevalent cancer worldwide, and the majority of cases are identified at an advanced stage, resulting in a disappointing five-year survival rate of 15% [1]. The treatment of choice for these patients includes chemotherapy, radiotherapy, targeted therapy, immunotherapy, or their combinations [1]. Somatic, activating mutations in the epidermal growth factor receptor (EGFR) gene are primarily located in exons 18 to 21, with deletions in exon 19 (exon 19 deletion) and codon 858 substitution in exon 21 (L858R) as the major ones [2]. Significant advances in the treatment and outcome of advanced EGFR-mutant lung cancer patients have been achieved with EGFR tyrosine kinase inhibitors (TKIs), especially for those patients with exon 19 deletion or L858R [3–5]. However, despite these favorable results, disease progression inevitably develops in virtually all patients after approximately 12 months of first- and second-generation TKI therapy [6]. Among the various mechanisms of acquired resistance to TKIs, development of the gatekeeper T790M mutation, a second site mutation at codon 790 in exon 20, is the most commonly encountered one, accounting for 50–60% of cases with acquired resistance to gefitinib, erlotinib, or afatinib [7–9].

The advent of the prototypic third-generation EGFR TKI, osimertinib, which is highly selective for both activating EGFR and T790M resistance mutations, offers further advantages to non–small cell lung cancer (NSCLC) patients with de novo or acquired T790M mutations [10–12]. Therefore, it is imperative to screen patients receiving first- or second-generation TKIs for the T790M mutation at the time of disease progression. Both plasma- and tissue-based screening approaches are recommended by guidelines and expert consensus statements for genomic resistance mechanisms at disease progression [13, 14]. Recently, the International Association for the Study of Lung Cancer (IASLC) adopted a “plasma first” approach for biomarker testing to identify mechanisms of resistance to targeted therapy, with tissue biopsy only if plasma circulating tumor DNA (ctDNA) is uninformative [15]. A variety of methodologies are available for ctDNA analysis, including both next-generation sequencing (NGS)- and polymerase chain reaction (PCR)-based methods. Although testing of plasma ctDNA by a validated NGS platform is preferred to single-gene, PCR-based approaches by the IASLC, limited PCR analysis for certain EGFR mutations may still have the advantages of low cost and comparable sensitivity [15].

In light of the importance of identifying the T790M mutation at disease progression in TKI-treated patients, several studies have investigated the prevalence of the T790M mutation and its predictors and prognostic impact [16–19]. However, most of the studies were cross-sectional and few, if any, collected a series of plasma samples to explore resistance mechanisms [20, 21]. Therefore, we designed a prospective longitudinal study to evaluate the development of the T790M mutation using droplet digital PCR (ddPCR) in NSCLC patients with activating EGFR mutations and treated with first-line TKIs. The trajectory and outcome of the patients, as well as their association with T790M status, were explored and discussed.

## Patients and methods

### Study design and participants

This prospective observational study was conducted at National Taiwan University Hospital in Taiwan. From April 2015 to June 2019, patients with systemic treatment-naïve, advanced lung adenocarcinoma, either newly diagnosed or recurrent after previous surgery, were screened for eligibility based on the following inclusion criteria: (1) aged 20 years or older; (2) presence of a tumor-harboring EGFR mutation, including exon 19 deletion, L858R, G719X, S768I, or L861Q; and (3) gefitinib, erlotinib, or afatinib as the treatment of choice at the discretion of the physician in charge. Patients were excluded from this study if they (1) had EGFR mutations other than those mentioned above; (2) had received TKIs other than the aforementioned three; or (3) were not willing to provide informed consent.

## Ethics

The authors are accountable for all aspects of the work in ensuring that questions related to the accuracy or integrity of any part of the work are appropriately investigated and resolved. The study was conducted in accordance with the Declaration of Helsinki (as revised in 2013). The study was approved by the research ethics committee of National Taiwan University Hospital (201304074RIPC) and informed consent was taken from all individual participants.

## Clinical management

Patients were assessed and treated at the discretion of the clinicians in charge. In our institution, all lung cancer patients underwent a complete staging workup, including brain, chest, and abdomen computed tomography (CT) and whole-body bone scintigraphy, or their alternatives, at the time of initial diagnosis. During the study period, gefitinib, erlotinib, and afatinib were reimbursed by Taiwan’s National Health Insurance (NHI) and were freely available to the clinicians. However, according to NHI regulations, patients should be reassessed for treatment response using chest CT and other image modalities at least every three months. Upon disease progression during TKI therapy, clinicians may choose to do a rebiopsy of the tumor tissue for further EGFR mutation analysis, based on their judgement. Tumor tissues were assayed using either the cobas EGFR Mutation Test v2 (Roche Diagnostics, Basel, Switzerland) [22], or matrix-assisted laser desorption ionization/time of flight mass spectrometry [23].

## Study procedures

After enrollment, blood samples were collected from the patients before commencement of TKI therapy. Thereafter, serial blood sampling was conducted at tri-monthly intervals, and the last blood sample was obtained at disease progression. An EDTA-containing venous blood collection tube was used for specimen acquisition. A total of 10 ml of blood was retrieved and processed to plasma within an hour as follows: The collection tube was centrifuged at 2,000 g for 10 min in a pre-chilled swing-out rotor at 4℃. The supernatant was carefully pipetted into a 15-ml Falcon tube, which was then centrifuged at 2,000 g for 10 min in a fixed angle rotor at 4℃. The plasma was again carefully pipetted off, transferred into the cryovial, and stored at -80℃ until ddPCR analysis in batch. Patients were followed up until the date of death or to the end of 2021.

## ddPCR

Liquid biopsy using ddPCR to detect the T790M mutation is an established technique [24–26], and this methodology in our laboratory has been granted LDTs (Laboratory Developed Tests) certification by the Taiwan FDA. Also, our laboratory was suitably equipped and proficient in the use of ctDNA to test for T790M mutation based on proficiency testing conducted by the Taiwan Society of Pathology and the Taiwan Society of Laboratory Medicine in 2021. The protocol for ddPCR-based liquid biopsy was as follows:

Frozen plasma samples were thawed at room temperature and extraction of ctDNA was performed using the QIAsymphony Virus/Bacteria kit (Qiagen, Valencia, CA, US), according to the manufacturer’s instructions. DNA was eluted in 70 µl of elution buffer and the volume was reduced to 10 µl by vacuum concentration. Genotyping of ctDNA for T790M detection was performed by a ddPCR EGFR Mutation Assay using the QX200 droplet digital PCR system (BioRad, Hercules, CA, US). In brief, the standard ddPCR master mix was assembled with a specific primer/probe mix (Applied Biosystems, Assay No.AHI1ZF4, Carlsbad, CA, US), ctDNA, and 2× ddPCR supermix (Bio-Rad, Cat. No.1,863,024). Samples were loaded into the DG8 cartridges (Bio-Rad, Cat. No.1,864,008) using 20 µl of the prepared ddPCR master mix, followed by 70 µl of droplet generation oil (Bio-Rad, Cat. No.1,863,005) in the adjacent wells. Each DG8 cartridge was placed into the QX200 droplet generator for emulsification, which produced about 20,000 droplets per sample. The generated droplets were then transferred onto a 96-well PCR plate (Bio-Rad, Cat. No.12,001,925) for 40 cycles of PCR. Following PCR amplification, the PCR plate was read with the QX200 Droplet Reader and the data were analyzed using the rare event detection (RED) mode of the QX200 analysis software (version 1.2.10.0, Bio-Rad). This mode is used for direct quantification of nucleic acid target sequences in cancer research. A fluorescence intensity threshold of 3,000 was set as a cutoff and a droplet above this threshold was scored as positive for T790M.

Experimental controls were also performed for each assay, including a no template control (NTC) for monitoring environmental contamination and a positive control (Multiplex I cfDNA Reference Standard Set, Cat. No.HD780, Horizon Discovery Ltd, Cambridge, UK) for confirmation of the assay performance.

In our laboratory, the limit of detection (LoD) for T790M was set at 0.23%. To calculate the LoD, we used commercially available cfDNA standards as test samples. In the test, two mutation rates of 0% (wild type) and 1% were used, and 20 detection reactions were performed for each mutation rate. The test results were substituted into the formulae below to obtain the limit of blank (LoB) and LoD. The test procedures were conducted based on the recommendations of Clinical and Laboratory Standards Institute (CLSI) EP17. The calculation formulae were as follows:

LoB = mean (0% sample) + 1.645 * standard deviation (0% sample).

LoD = LoB + 1.645 * standard deviation (1% sample).

## Data collection

We collected patient demographics, Eastern Cooperative Oncology Group (ECOG) performance status [27], smoking history, extent of cancer spread, clinical staging based on the 7th American Joint Committee on Cancer staging system,[28] EGFR mutation types, and the initial choice of TKI at the time of therapy commencement. Then, data on the best response to first-line TKI therapy on the basis of the RECIST criteria (version 1.1) [29], duration of TKI use, time to disease progression, subsequent second-line treatment after failure of TKI therapy, and overall survival from the initiation date of TKI therapy were obtained, along with the clinical course of the patients. Where available, the results of rebiopsy in terms of T790M status were also retrieved.

### Statistical analysis

Descriptive statistics are presented as medians (interquartile ranges), frequency distribution, and percentages. The Pearson’s χ^2^, Fisher’s exact, Mann-Whitney U, and Kruskal-Wallis H tests were used as statistical methods. The Kaplan-Meier curves were used to analyze time-to-event data. Statistical significance was set as P < 0.05. All statistical analysis was performed using the SPSS 20.0 software package (SPSS, Inc., Chicago, IL, US).

## Results

### Study population

**Fig. 1:**
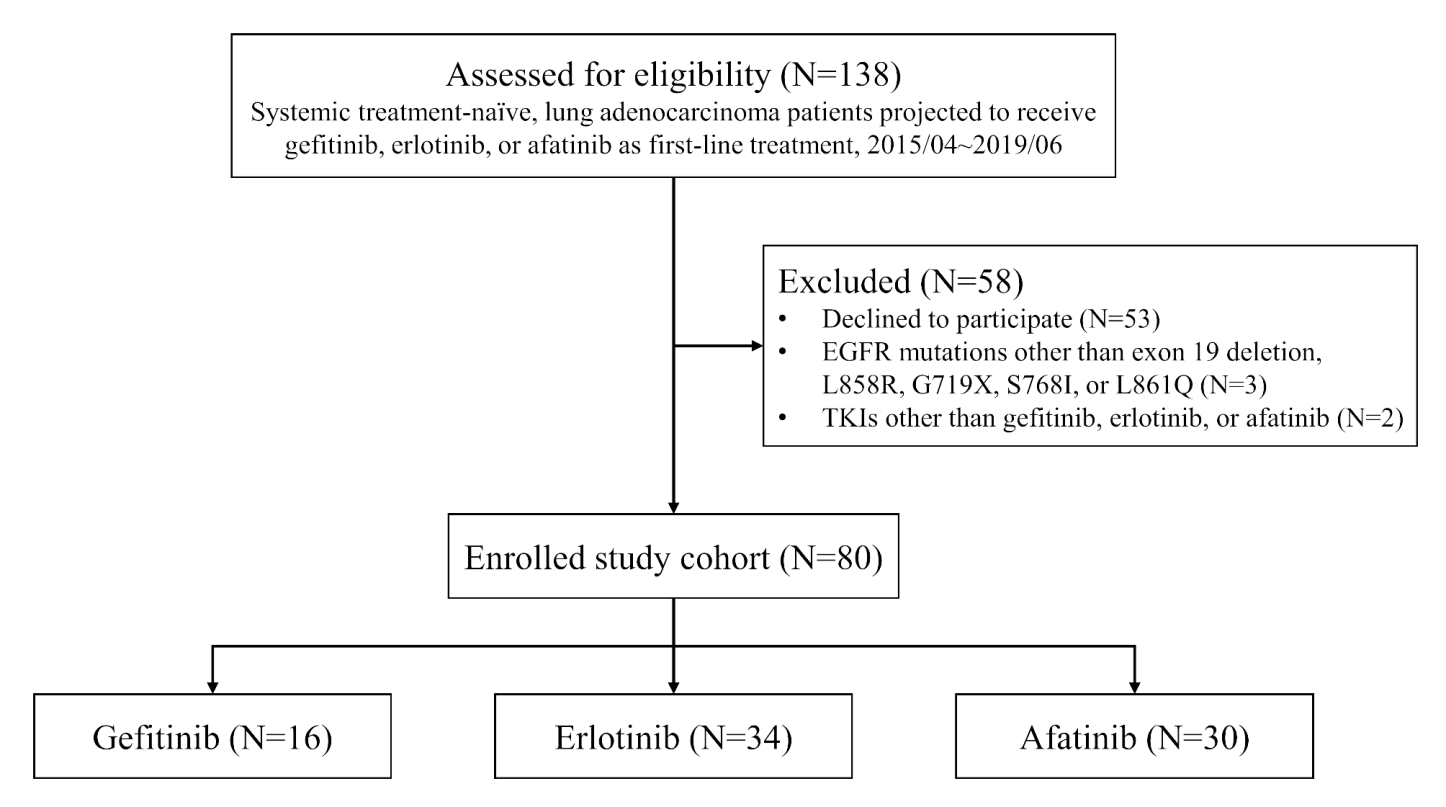
Study flow diagram EGFR, epidermal growth factor receptor; TKI, tyrosine kinase inhibitor

A total of 80 lung adenocarcinoma patients were included during the study period (Fig. 1). The average age of the study cohort was 64.5 years, and 34 (43%) of the 80 patients were male (Table 1). The majority of the patients had an ECOG of 0–1 and stage IIIB or IV disease. The driver gene alterations identified in the cohort were EGFR exon 19 deletion (N = 41), L858R (N = 35), L861Q (N = 2), G719X (N = 1), and L858R + S768I (N = 1). Fifty-five (69%) of the patients achieved a partial response and 21 (26%) had stable disease as their best response to first-line TKIs. At the time of data cutoff, the median time to progression, duration of first-line TKI, and overall survival of the study participants were 13.8 (8.2–21.6), 18.5 (11.1–26.1), and 36.2 (23.3–54.9) months, respectively. Five patients had not experienced progressive disease and were still receiving TKI therapy at the time of data cut-off (December 2021), and 28 patients were still alive at that time.

**Table 1:** Baseline characteristics of the study population (N = 80)

Characteristics	Total cohort (N = 80)	Gefitinib (N = 16)	Afatinib (N = 30)	Erlotinib (N = 34)	P values
Age, y	64.5 (57.2–70.5)	67.0 (62.5–79.0)	60.5 (54.3–66.0)	65.0 (56.8–69.5)	0.019
Male sex	34 (43)	8 (50)	11 (37)	15 (44)	0.663
ECOG					
0–1	74 (93)	14 (88)	27 (90)	33 (97)	0.393
≥2	6 (8)	2 (13)	3 (10)	1 (3)	
Smoking status					
Current or former smoker	23 (29)	6 (38)	8 (27)	9 (27)	0.688
Never-smoker	57 (71)	10 (63)	22 (73)	25 (74)	
Stage					
IIIB/IV	73 (91)	14 (88)	27 (90)	32 (94)	0.708
Post-operative recurrence	7 (9)	2 (13)	3 (10)	2 (6)	
EGFR mutation					
L858R	35 (44)	9 (56)	11 (37)	15 (44)	0.095
Exon 19 deletion	41 (51)	7 (44)	15 (50)	19 (56)	
Others†	4 (5)	0 (0)	4 (13)	0 (0)	
Initial presentation					
Malignant pleural effusion	25 (31)	5 (31)	8 (27)	12 (35)	0.759
Malignant pericardial effusion	3 (4)	0 (0)	1 (3)	2 (6)	0.587
Brain metastasis	29 (36)	2 (13)	8 (27)	19 (56)	0.005
Liver metastasis	7 (9)	2 (13)	1 (3)	4 (12)	0.412
Bone metastasis	40 (50)	7 (44)	13 (43)	20 (59)	0.398
Best response to EGFR TKI					
Partial response	55 (69)	9 (56)	24 (80)	22 (65)	0.522
Stable disease	21 (26)	6 (38)	5 (17)	10 (29)	
Progressive disease	4 (5)	1 (6)	1 (3)	2 (6)	
Time to progression, mo‡	13.8 (8.2–21.6)	14.3 (9.8–25.2)	15.8 (7.2–24.2)	13.3 (7.3–19.0)	0.429
Duration of first-line TKI, mo§	18.5 (11.1–26.1)	22.7 (12.5–31.2)	21.0 (12.0–28.7)	15.1 (9.0–23.6)	0.144
Overall survival, mo¶	36.2 (23.3–54.9)	49.5 (35.5–56.1)	37.5 (23.6–58.1)	33.0 (20.5–40.9)	0.043

ECOG, Eastern Cooperative Oncology Group; EGFR, epidermal growth factor receptor; TKI, tyrosine kinase inhibitor

† G719X, L861Q, and L858R + S768I.

‡ No disease progression was observed in 5 patients at the time of data cutoff (December 2021)

§ In December 2021, 5 patients were still receiving TKI therapy

¶ A total of 28 patients remained alive at the end of 2021

Erlotinib (N = 34) was the most frequently prescribed first-line TKI in this study (Table 1), followed by afatinib (N = 30) and gefitinib (N = 16). Patients receiving afatinib were younger than those receiving gefitinib. The frequency of initial brain metastasis was higher in patients treated with erlotinib than in those treated with gefitinib or afatinib. The overall survival of patients who received gefitinib treatment was longer than that of those who received erlotinib treatment.

## T790M mutation status at progression

At the end of 2021, 75 of the 80 enrolled patients experienced progressive disease, and 53 (71%) of them underwent rebiopsy. The reasons for not having rebiopsy performed included inaccessible site of rebiopsy (N = 6), patient refusal (N = 5), decline in ECOG performance status (N = 4), physician discretion (N = 4), and rapid progression (N = 3). The T790M mutation was identified in 53% (28/53) of the rebiopsy samples. Meanwhile, plasma ddPCR detected a T790M mutation in 23 (43%) out of 53 patients at disease progression (Table 2). The concordance rate between ddPCR and rebiopsy was 76% with regard to T790M, and ddPCR recognized an additional 4 (8%) T790M-positive patients. Among the 22 patients who did not receive rebiopsy, 10 (45%) tested positive for T790M mutation by ddPCR.

**Table 2:** Contingency table showing test results of rebiopsy and plasma ddPCR for EGFR T790M mutation (N = 53)

	Rebiopsy T790M	
ddPCR T790M	Positive	Negative
Positive	19 (36)	4 (8)
Negative	9 (17)	21 (40)

Taken together, at the time of disease progression, 56% (42/75) of the patients harbored a T790M mutation, as detected by combined ddPCR and rebiopsy. In this study, comparisons between patients with and without a T790M mutation at progression showed no specific features associated with the development of acquired T790M-dependent resistance to first-line EGFR TKIs (Table 3).

**Table 3:** Characteristics of patients with regard to T790M mutation status at disease progression (N = 75)

Characteristics	T790M(-) (N = 33)	T790M(+) (N = 42)	P values
Age, y	65.0 (58.5–73.5)	63.5 (56.0–68.3)	0.313
Male sex	14 (42)	17 (41)	0.865
ECOG			
0–1	30 (91)	39 (93)	0.999
≥2	3 (9)	3 (7)	
Smoking status			
Current or former smoker	7 (21)	14 (33)	0.246
Never-smoker	26 (79)	28 (67)	
Stage			
IIIB/IV	30 (91)	38 (91)	0.999
Post-operative recurrence	3 (9)	4 (10)	
EGFR mutation			
L858R	15 (46)	18 (43)	0.672
Exon 19 deletion	16 (49)	23 (55)	
Others†	2 (6)	1 (2)	
First-line EGFR TKI			
Gefitinib	7 (21)	8 (19)	0.472
Erlotinib	12 (36)	21 (50)	
Afatinib	14 (42)	13 (31)	
Initial presentation			
Malignant pleural effusion	11 (33)	14 (33)	0.999
Malignant pericardial effusion	0 (0)	3 (7)	0.251
Brain metastasis	11 (33)	16 (38)	0.670
Liver metastasis	1 (3)	6 (14)	0.126
Bone metastasis	18 (55)	21 (50)	0.696
Best response to EGFR TKI			
Partial response	21 (64)	30 (71)	0.420
Stable disease	9 (27)	11 (26)	
Progressive disease	3 (9)	1 (2)	
Time to progression, mo	12.4 (7.1–19.9)	13.7 (7.9–20.0)	0.898
Duration of first-line TKI, mo	17.3 (10.1–25.4)	17.0 (10.7–24.5)	0.709
Overall survival, mo‡	35.5 (23.8–56.0)	36.2 (22.0–52.0)	0.572

ECOG, Eastern Cooperative Oncology Group; EGFR, epidermal growth factor receptor; TKI, tyrosine kinase inhibitor

† G719X, L861Q, and L858R + S768I.

‡ A total of 23 patients remained alive at the end of 2021

## Association of detected plasma T790M with disease progression

At disease progression, a T790M mutation was detected by ddPCR in 33 (44%) of the 75 patients and the median T790M copy number of these patients was 10.4 (5.6–53.5) copies/ml. Longitudinal analysis of plasma ddPCR for T790M was carried out with these patients. The median time to detection of plasma T790M was 9.5 (5.6–15.1) months, and emergence of T790M was always associated with disease progression, which was later documented based on clinical assessment and radiological investigation. The median time interval from the presence of T790M in the plasma samples to physician-defined progression was 1.1 (0–4.1) months. In our cohort, early progression as indicated by the plasma T790M mutation could be detected up to 16.6 months earlier than that detected by radiological progression.

## Impact of T790M mutation on overall survival

**Fig. 2:**
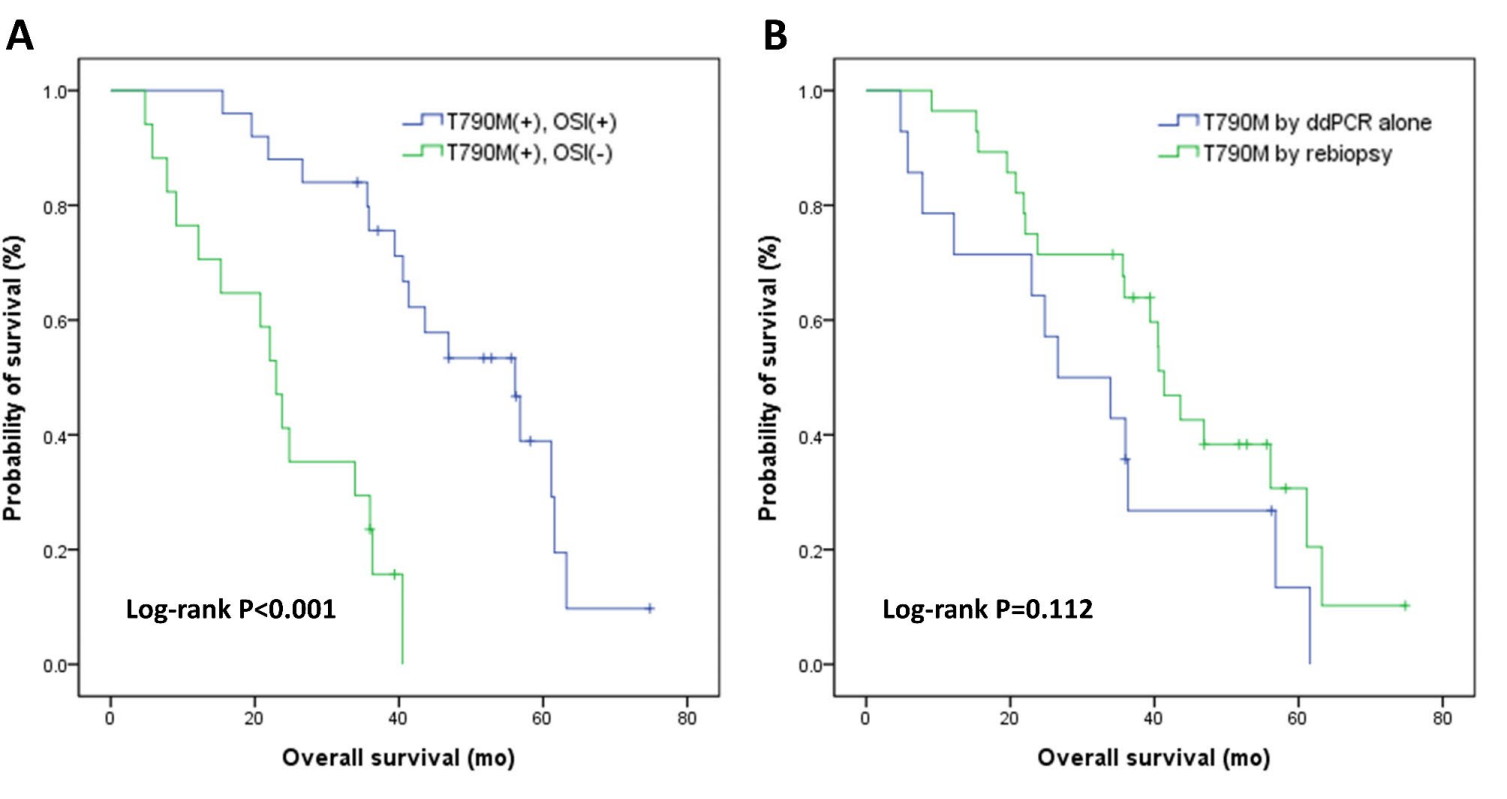
Kaplan-Meier survival curves of overall survival analysis in (A) patients harboring a T790M mutation at progression, with (N = 25) and without (N = 17) second-line osimertinib; and (B) patients with a T790M mutation detected by rebiopsy (N = 28) or by plasma ddPCR alone (N = 14) OSI, osimertinib

After disease progression, 25 (60%) of the 42 patients with a T790M mutation received osimertinib as second-line TKI therapy. The median overall survival of the patients who received osimertinib was longer than that of those who did not (Fig. 2A). Among the 14 patients with T790M detected solely by ddPCR, the median overall survival was 26.6 (9.9–43.3) months; only 4 (29%) patients were prescribed osimertinib (Fig. 2B), because rebiopsy was not conducted or was not informative. In comparison, 28 patients with rebiopsy showing T790M had a median overall survival of 41.3 (36.6–46.0) months, and three-fourths of them (21/28) had received osimertinib.

A total of 17 patients with T790M detected by ddPCR were treated with osimertinib after disease progression and their median T790M copy number was 9.3 (4.8–58.0) copies/ml. Treatment responses to osimertinib in 8 patients with T790M copy numbers ≥ 9.3 copies/ml were 5 partial response (63%), 2 stable disease (25%), and 1 progressive disease (13%). Among 9 patients with T790M copy numbers < 9.3 copies/ml, 3 (33%) and 6 (67%) patients had partial response and stable disease, respectively, in response to osimertinib treatment. The median survivals for patients with T790M copy numbers ≥ 9.3 vs. <9.3 copies/ml were 42.5 (30.1–53.8) vs. 55.6 (41.3–61.4) months (Log-rank P = 0.435).

## Discussion

The main findings of this prospective observational study are as follows: (a) in lung adenocarcinoma patients with sensitizing EGFR mutations, treated with first-line TKIs, the disease control rate was 95% (76/80), and the median time to progression, duration of first-line TKIs, and overall survival were 13.8, 18.5, and 36.2 months, respectively; (b) gefitinib-treated patients had longer overall survival than erlotinib-treated patients, and the former patients also had a significantly lower proportion of initial brain metastasis than the latter patients; (c) at disease progression, the concordance rate with regard to T790M between ddPCR and rebiopsy was 76% in 53 patients; (d) T790M mutation was detected by ddPCR in 33 (44%) of the 75 patients at progression and 14 of them had T790M identified only by ddPCR; (e) emergence of plasma T790M was always associated with disease progression, and the median time interval from the presence of T790M in plasma to progression was 1.1 (0–4.1) months; (f) a little more than one-fourth of patients who had T790M detected solely by ddPCR received osimertinib; in comparison, three-fourths of patients with rebiopsy showing T790M were prescribed osimertinib. Taken together, our study suggests that serial assessment of plasma for T790M using ddPCR is beneficial for lung cancer patients on first-line TKIs in terms of early detection of this dominant molecular resistance mechanism. Furthermore, early detection may facilitate prescription of osimertinib to appropriate candidates, especially in cases in which tissue rebiopsy is not feasible or is uninformative.

The median overall survival in our cohort was 36.2 months, comparable to a recent report of 37.0 months in advanced EGFR-mutant NSCLC patients receiving gefitinib, erlotinib, or afatinib [19], as well as another report of 36.7 months in those treated with afatinib alone in our institution [30]. Moreover, our median time to progression was 13.8 months, consistent with that (12.4–14.4 months) of two other studies [19, 30], indicating the representativeness of our study cohort. Of note, overall survival in these real-world data is seemingly longer than that in clinical trials [31, 32]. In the LUX-Lung 7, the median overall survival for afatinib and gefitinib was 27.9 and 24.5 months, respectively [32]. The phase III CTONG 0901 also reported similar overall survival of 22.9 vs. 20.1 months for erlotinib vs. gefitinib [31]. These findings may highlight the importance of physician involvement and personalized, tailored care for cancer patients beyond guidelines to yield a better clinical outcome.

The T790M mutation confers resistance to first-line TKIs via sterically lowering their affinity to the ATP binding pocket [8]. Since patients harboring an acquired T790M mutation benefit most from second-line Osimertinib [11], it is imperative to identify the presence of this mutation at disease progression while on first- or second-generation TKIs. Combined ddPCR and rebiopsy in the current study found that T790M developed in 56% of our patients at progression, a rate that falls between the 46% and 66% reported in the literature [9, 18, 33–35]. Some studies have shown a number of features associated with the occurrence of a secondary T790M mutation [9, 19, 36–42]. Patients with exon 19 deletion were reportedly more likely to develop T790M compared to other mutations [36, 37]; however, patients treated with afatinib did not acquire T790M as often as those treated with gefitinib or erlotinib [37, 40–42]. Although our study was not able to detect statistically significant differences in patient characteristics between the T790M-positive and -negative groups due to limited case numbers, our results basically fit previous findings. For instance, a higher proportion of our patients with exon 19 deletion developed a T790M mutation compared to those with L858R (23/39 [59%] vs. 18/33 [55%]). Also, afatinib-treated (13/27, 48%) patients in this cohort were less likely to test positive for a T790M mutation than gefitinib- (8/15, 53%) or erlotinib-treated (21/33, 64%) patients. In spite of these proposed predictors for a secondary T790M mutation, rebiopsy remains the standard of care to allow detection of the gatekeeper mutation [15]. Therefore, it may be more practical and attractive to have a reliable, cost-effective, and non-invasive method for assessment of T790M resistance mutation. In addition, patient education is an important acceptance facilitating intervention on rebiopsy. Access to available and affordable third-generation EGFR TKIs may also be the key to increase the rebiopsy rate.

Single-gene testing for T790M using plasma ddPCR is clinically applicable and promising for the selection of patients who have progressed during first-line TKI therapy for treatment with Osimertinib [43, 44]. It is not surprising that our study clearly showed the survival advantage of T790M-positive patients treated with osimertinib compared to those without. However, given the limited utilization of rebiopsy, only 28 (67%) out of 42 patients were identified with T790M at disease progression, using both plasma ddPCR and rebiopsy as the gold standard. Plasma ddPCR detected 33 (79%) T790-positive patients; among those, 14 (33%) had a T790M mutation detected solely by ddPCR. Moreover, there are some concerns about performing rebiopsy, such as difficulty in accessing recurrence sites, and patient refusal of invasive procedures [45, 46]. Taken together, plasma ddPCR may serve as a useful alternative or adjunct to rebiopsy while tailoring subsequent treatment for patients progressing on first- or second-generation TKIs. However, if T790M is not detected by plasma ddPCR, checking of T790M status using ddPCR or other detection assays in the tissue sample should still be considered since the sensitivity of plasma T790M testing by ddPCR is reportedly around 80% with the tissue test result as the reference [47, 48].

In our study, three-fourths, but not all, of the patients with rebiopsy showing T790M were prescribed osimertinib because this third-generation EGFR TKI was not reimbursed by the NHI in Taiwan during the study period. On the contrary, given that the physicians in charge were blinded to the ddPCR results, only a little more than one-fourth of patients who had T790M detected solely by ddPCR received osimertinib. These patients may be empirically treated with osimertinib on disease progression according to the available evidence from the AURA study demonstrating its effectiveness regardless of acquired T790M resistance [12].

In addition, owing to the ease of serial sampling, ctDNA is emerging as the preferred method for real-time monitoring of resistance mutations [15]. In line with previous studies [21, 49], our patients with detectable plasma T790M eventually experienced disease progression. In the present study, the T790M resistance mechanism was detected 1.1 months before objective disease progression, at a later time point than in the study by Kim and associates [21], which found molecular progressive disease 3.4 months prior to clinical progression. The discrepancy may be explained by different follow-up schedules (i.e., tri-monthly in our study vs. monthly or bi-monthly in Kim’s) [21]. Timely cancer treatment is of paramount importance; thus, serial follow-up of plasma ddPCR for T790M may herald disease progression and should prompt clinical assessment in patients on first-line TKIs for sensitizing mutations.

We do acknowledge a few limitations pertaining to this study. First, it was performed in a single center and enrolled a limited number of patients; thus, the generalizability of the results could be limited. Nonetheless, our patient characteristics were similar to those in a multicenter study in Taiwan in terms of age, gender, and EGFR mutations [9]. Second, although there are institutional guidelines for NSCLC patients, the physicians in charge may not be fully compliant with the guideline recommendations, thus compromising the standardization of patient care. However, compared with randomized controlled trials [31, 32], the longer overall survival observed in our cohort highlights the importance of real-world patient selection and treatment decisions. Third, the FLAURA trial revealed the survival advantage with first-line osimertinib compared to gefitinib or erlotinib in patients with EGFR exon 19 deletion or L858R mutations [50], leading to the approval of osimertinib as first-line therapy. In this regard, we would argue that single-gene ddPCR for T790M, as reported in the current study, is becoming less relevant as osimertinib is moving into a frontline setting. However, accessibility to first- or even second-line osimertinib remains limited in certain countries, such as Taiwan, due to an economic barrier or health insurance regulations. Therefore, our study findings are still clinically relevant and valuable for healthcare providers in caring for lung cancer patients.

## Conclusion

In summary, our understanding of human disease, including cancer, has shifted the paradigm towards personalized/precision medicine. With regard to lung cancer, the advent of EGFR TKIs has revolutionized the treatment of EGFR-mutant NSCLC and significantly improved the prognosis of these patients. Our study demonstrates that ddPCR-based plasma genotyping is a technology of great utility for the detection and monitoring of a key actionable genomic alteration, namely, T790M, in patients on gefitinib, erlotinib, or afatinib for activating mutations. In line with the plasma-first approach by guideline recommendations, we suggest that plasma single-gene ddPCR may substitute for or supplement traditional tissue rebiopsy to achieve better patient care and outcome when plasma NGS is not available.

## Data Availability

The datasets used and/or analysed during the current study are available from the corresponding author on reasonable request.

## List of abbreviations

CI: confidence interval
CT: computed tomography
ctDNA: circulating tumor
ddPCR: droplet digital PCR
ECOG: Eastern Cooperative Oncology Group
EGFR: epidermal growth factor receptor
IASLC: International Association for the Study of Lung Cancer
NGS: next-generation sequencing
NHI: National Health Insurance
NSCLC: non–small cell lung cancer
PCR: polymerase chain reaction
TKI: tyrosine kinase inhibitor
